# Epigenetic Control of Gonadal Aromatase (*cyp19a1*) in Temperature-Dependent Sex Determination of Red-Eared Slider Turtles

**DOI:** 10.1371/journal.pone.0063599

**Published:** 2013-06-07

**Authors:** Yuiko Matsumoto, Alvin Buemio, Randy Chu, Mozhgon Vafaee, David Crews

**Affiliations:** Section of Integrative Biology, University of Texas at Austin, Austin, Texas, United States of America; Massachusetts General Hospital, United States of America

## Abstract

In the red-eared slider turtle (*Trachemys scripta*), a species with temperature-dependent sex determination (TSD), the expression of the aromatase gene during gonad development is strictly limited to the female-producing temperature. The underlying mechanism remains unknown. In this study, we identified the upstream 5′-flanking region of the aromatase gene, gonad-specific promoter, and the temperature-dependent DNA methylation signatures during gonad development in the red-eared slider turtle. The 5′-flanking region of the slider aromatase exhibited sequence similarities to the aromatase genes of the American alligator, chicken, quail, and zebra finch. A putative TATA box was located 31 bp upstream of the gonad-specific transcription start site. DNA methylation at the CpG sites between the putative binding sites of the fork head domain factor (FOX) and vertebrate steroidogenic factor 1 (SF1) and adjacent TATA box in the promoter region were significantly lower in embryonic gonads at the female-producing temperature compared the male-producing temperature. A shift from male- to female-, but not from female- to male-, producing temperature changed the level of DNA methylation in gonads. Taken together these results indicate that the temperature, particularly female-producing temperature, allows demethylation at the specific CpG sites of the promoter region which leads the temperature-specific expression of aromatase during gonad development.

## Introduction

Temperature-dependent sex determination (TSD) is an example of developmental plasticity where the ambient temperature of the embryos leads to the development of the bipotential gonads into either testes or ovaries. In TSD species such as the American alligator and some turtle species, gonadal development can be modified by the concentration of sex steroid hormones in the surrounding system of the embryos. For example, an excess of either testosterone or estrogen during gonadal differentiation leads to testicular or ovarian development, respectively, regardless of the surrounding temperature [1]–[3]. In non-mammalian vertebrates with genotypic sex determination (GSD) such as fish and amphibians, the manipulation of the embryonic environment with sex steroid hormones often overrides the gonadal trajectory preset by genotypes, which leads to a skewed sex ratio at hatching [4]–[6]. Cytochrome P450 aromatase, also known as *cyp19a1* or aromatase, is the enzyme that irreversibly catalyzes androgens into estrogens, and therefore plays a central role in balancing the production of steroid hormones. Elevated expression of the aromatase gene is consistently observed in differentiating ovaries among taxa while its expression is usually suppressed during development of testes [7]–[9]. Blocking aromatase activity with aromatase inhibitors during gonad development also results in a redirection of the putative gonadal sex usually determined by genetic- or environmental- signals in birds, reptiles, and fish [10]–[12]. Therefore, aromatase is considered to be the essential factor in determining gonad fate during development in non-mammalian vertebrates.

In the red-eared slider turtle (*Trachemys scripta*) the level of aromatase expression in gonads depends on the ambient temperature; high expression is exhibited at female-producing temperature (FPT, 31°C) while low expression is observed at male-producing temperature (MPT, 26°C) [13]. The increase in aromatase expression in embryonic gonads incubating at a FPT coincides with the temperature-sensitive period (TSP) of development where embryonic gonads are sensitive to surrounding temperature [1], [13]. A drastic change in gonad aromatase expression is also observed during the temperature-induced change in sex ratio in fish and amphibians [9], [14], [15]. These findings suggest that temperature regulates the transcription of aromatase gene, resulting in the differentiation of bipotential gonads to a particular gonad phenotype. In species with TSD, however, the sequence and regulatory mechanisms of the 5′-flanking region of aromatase gene remain mostly unclear.

Accumulating evidence suggests that epigenetic modification such as histone modification and DNA methylation mediate specific gene expression changes in response to the environment during development [16], [17]. Particularly, DNA methylation levels at the regulatory regions are negatively correlated with the level of gene expression and often regulate the temporal and spatial expression of development-related genes [18]–[21]. Recent evidence in species with GSD with temperature influences has linked the environment, particularly temperature-related regulation of aromatase expression, to DNA methylation in the aromatase promoter region [22]. The developmental patterns of DNA methylation pertinent to aromatase gene expression that may be directed by temperature have never been investigated in species with TSD. We identified the gonad-specific promoter and upstream 5′-flanking region of the aromatase gene and found the temperature-specific pattern of DNA methylation on the putative promoter region during gonad development in the red-eared slider turtle.

## Results

### The 5′ upstream region of translation start codon are highly conserved among species

**Figure 1:**
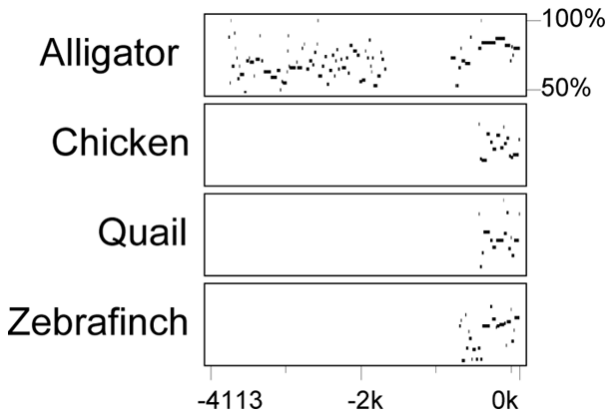
Sequence homology analysis of the 5′-flanking region of the aromatase in the red-eared slider turtle. The newly identified 4113 bp sequence of the aromatase in the red-eared slider turtle (GenBank accession no. KC554066), consisting of 4066 bp upstream and 47 bp downstream of the translation start codon (ATG), was pair-aligned with the aromatase sequences (upstream bp length from ATG/total bp sequence compared) of the American alligator (3950/4000), chicken (2614/3229), quail (2442/4351), and zebra finch (1356/1401) using the percentage identity plot (PIP) Maker [24]. GenBank accession numbers are as follows: *alligator* (AY029233), *chicken* (D50335), *quail* (D50336), and *zebra finch* (AH008871). The additional 5′ flanking region of alligator aromatase was extracted from the assembled genome [68]. The horizontal axis represents the base-pair position of the aromatase sequence of the red-eared slider turtle, and the vertical axis represents the percent nucleotide similarity of each aligned segment (i.e., stretch of DNA without any gaps) with the corresponding species.

We identified and sequenced a novel 3944 bp of 5′-flanking region of the aromatase gene. A total sequence of 4066 bp was revealed upstream from the translation start codon (ATG), which was previously identified in the red-eared slider turtle [23]. We compared sequence homology of this 5′-flanking region of slider aromatase to other species using Percent identity plot (PIP) [24]. In this analysis, the 4113 bp of slider aromatase 5′-flanking region near the ATG was pair-aligned with the aromatase gene sequence of four different species: American alligator (*Alligator mississippiensis*), chicken (*Gallus gallus*), quail (*Coturnix coturnix*), and zebra finch (*Taeniopygia guttata*). These species were selected because of their sequence similarities in the aromatase 5′-flanking region to the slider aromatase according to a nucleotide BLAST search (National center for biotechnology information, Bethesda, MD). Homology analysis showed that the 5′-flanking sequence was particularly conserved the region within the approximately 800 bp upstream of the ATG (Figure 1). The American alligator, another species with TSD, exhibited the most number of nucleotide matches throughout the compared region. Comparisons with bird species showed that the zebra finch had the most number of nucleotide matches to the slider aromatase (Figure 1).

### Gonad-specific transcription start site is identified at 122 bp upstream of the translation start codon

The gonad-specific transcription start site (TSS) of aromatase was identified on mRNAs that were purified from embryonic gonads at stage 16, 19, and 21 at FPT. During these stages, the expression of aromatase exhibits a rapid increase in the embryonic gonads that is specific to FPT [13]. At stage 16, two distinct TSSs were found at 122 bp and 190 bp upstream of the ATG (Figure S1A). At stage 19 and 21, however, most clones exclusively produced mRNA that contained the start site at the 122 bp position (Figure S1A). Therefore, we re-designated the site as +1, the first nucleotide guanine, as gonad-specific TSS for subsequent studies.

### Putative transcription factor binding sites for conserved factors in vertebrate sex determination are found on the 5′- flanking region of aromatase

**Figure 2:**
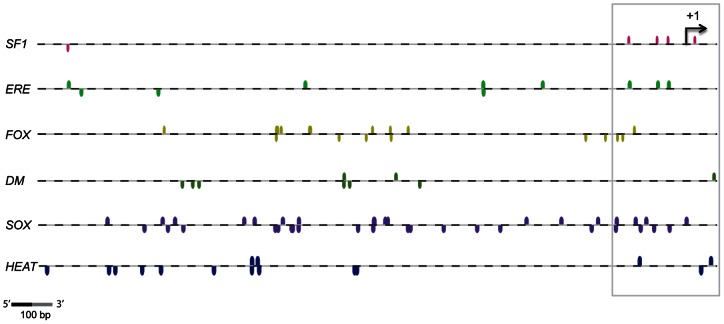
Prediction of transcription factor binding sites (TFBSs) on the 5′-flanking region of aromatase. TFBSs were predicted using the MatInspector (Genomatix). Colored marks represent the TFBSs with a 5′ to 3′ (above the line) or a 3′ to 5′ (below the line) orientation relative to the transcription start site (TSS). SF1: vertebrate steroidogenic factor 1, ERE: estrogen response elements, FOX: fork head domain factors, DM: DM domain-containing transcription factor, SOX: Sox/SRY sex/testis-determining and related HMG box factors, HEAT: heat shock factors. Horizontal arrow represents TSS (+1). A magnification of gray box is shown in Figure 3.

Next, we identified the putative transcription factor binding sites (TFBSs) on the total 4113 bp of the 5′-flanking region of aromatase sequence. Previous studies show that the following transcription factors have conserved roles in vertebrate sex determination and exhibit temperature-specific expression patterns in slider gonads during TSP: steroidogenic factor 1 (Sf1), estrogen receptor (ER), forkhead box protein L2 (FoxL2), doublesex and mab-3 related transcription factor 1 (Dmrt1), and SRY-box 9 (Sox9) [13], [25]–[27]. Therefore, we examined the corresponding binding sites of SF1, ERE, FOX, DM, and SOX respectively within the 5′-flanking region of the aromatase gene. In addition, accumulating evidence suggest that heat shock factors can directly modify the expression of development-related genes besides their original role in regulating heat shock genes [28], [29]. Thus, the binding site for heat shock factor (HEAT) was also included in the analysis. We show that the binding sites for all factors were predicted within the 5′-flanking region of the slider aromatase sequence. Interestingly, the locations of these binding sites were uniquely distributed in this upstream region. SF1 binding sites were mostly located around the region of TSS and often overlapped with ERE (Figure 2) due to their sequence similarities [30]. FOX- and DM-domain sites tended to be clustering closely (i.e., multiple binding sites within 100 bp) whereas SOX sites were periodically distributed throughout the region (Figure 2). The HEAT binding site was mainly located at either the 5′ or 3′ ends of this flanking region (Figure 2). No CpG island was detected within this region (data not shown).

We also compared the pattern of TFBSs within the homologous sequence region among species (Figure S2). Approximately 700 bp of the 5′-flanking sequence from the slider turtle and four other species were aligned relative to their TSSs. Others have noted that the chicken and quail aromatase TSS were ovarian-derived while the zebra finch aromatase TSS was identified as brain-specific [31]–[33]. Our comparative analysis detected conserved binding sites of SF1/ERE and SOX that were located around the −150 to −100 bp region in the turtle, chicken, and zebra finch (solid arrows in Figure S2). The quail sequence did not follow this pattern, though both the quail and chicken aromatase sequences showed a shared pattern of ERE and FOX binding sites just prior to the TSS. The slider aromatase sequence contained two SOX binding sites at approximately −300 bp that were located in a similar pattern as observed in the chicken and quail sequences (dot arrows in Figure S2).

### DNA methylation pattern in the aromatase promoter depends on incubation temperature

**Figure 3:**
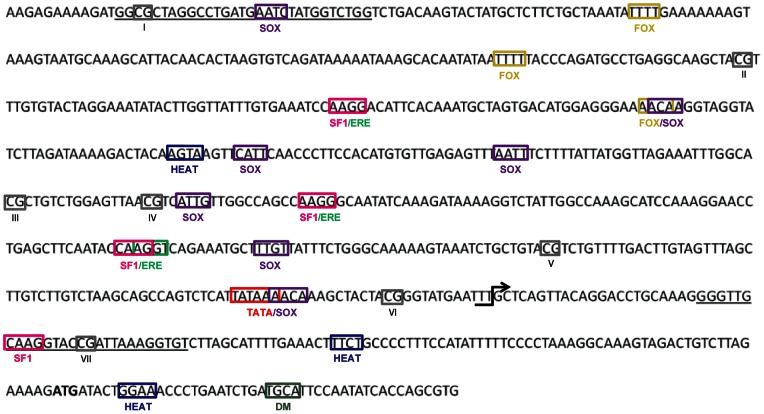
Putative aromatase promoter (−583 to +170) sequence in the gonads. The core conserved sequences (four nucleotides) for transcription factor binding sites (TFBSs) and CpG sites (I – VII) are indicated in colored and gray boxes, respectively. The gonad-specific transcription start site (TSS, +1) is indicated with an arrow. The translation initiation codon, ATG, is indicated in bold. The sequences of the Arom Set5 primer pairs used for DNA methylation analysis are underlined. SF1: vertebrate steroidogenic factor 1. ERE: estrogen response elements. FOX: fork head domain factors. DM: DM domain-containing transcription factor. SOX: Sox/SRY sex/testis-determining and related HMG box factors. HEAT: heat shock factors. TATA: TATA box.

DNA methylation level at the regulatory regions often regulates the temporal and spatial expression of development-related genes [20], [21]. We examined if the DNA methylation signature within the putative promoter region was different depending on incubation temperature in the slider turtle gonad. First, the region around the gonad-specific TSS was examined closely in the aromatase gene (a gray box in Figure 2). We found the TATA box located at −31 bp relative to the TSS, adjacent to the SOX binding sites (Figure 3). At the upstream of the TATA box, several FOX, SF1/ERE, SOX binding sites were located. HEAT binding sites were found before and after the TSS, and one DM site was found after the TSS. Based on the presence of the TATA box upstream of the gonad-specific TSS, we defined this 5′-flanking region as the putative promoter of aromatase in the differentiating ovaries.

**Figure 4:**
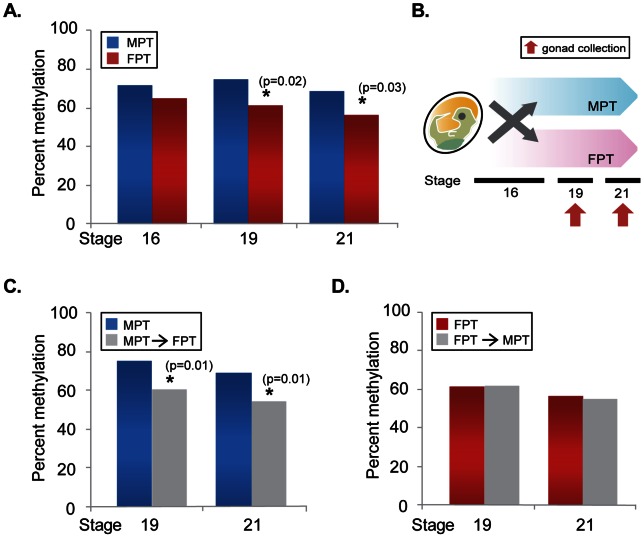
Total DNA methylation level of the putative aromatase promoter in the gonads. Total percent DNA methylation during embryonic development at MPT and FPT (A), MPT shifted to FPT (C), and FPT shifted to MPT (D) were examined. (B) The scheme of temperature-shift experiment. Each bar represents twenty to twenty-two clones/stage/temperature from the data of a single pool of 10 gonads. Asterisk (*) is statistically significant (p<0.05) according to the Fisher's exact test. MPT: male-producing temperature. FPT: female-producing temperature.

Second, we constructed Arom Set5 primers to amplify the promoter region containing 7 CpG sites that are potential targets for DNA methylation (Figure 3) and examined DNA methylation level. At stage 16, there was no difference in overall percentage of DNA methylation in the putative promoter at two temperatures in the gonads; however, DNA methylation levels were significantly lower at FPT during stage 19 and 21 compared to MPT (Figure 4A). To test the hypothesis that temperature acts by establishing DNA methylation signatures during the TSP, eggs were shifted to the opposite temperature (i.e., MPT→FPT or FPT→MPT) at stage 16, and methylation status examined at stages 19 and 21 (Figure 4B for experimental scheme). Previous studies show that temperature-shifts at stage 16 completely change the gene expression pattern and subsequent gonad trajectory, producing the gonad phenotype corresponding to the shifted temperature in red-eared slider turtles [1], [13], [34]. We found that the overall percentage of DNA methylation was significantly lower in MPT→FPT gonads compared to the control MPT (Figure 4C). This suggests that demethylation is allowed by temperature change to FPT after stage 16. However, there was no difference in the overall percentage of DNA methylation between FPT→MPT and the control FPT groups (Figure 4D).

**Figure 5:**
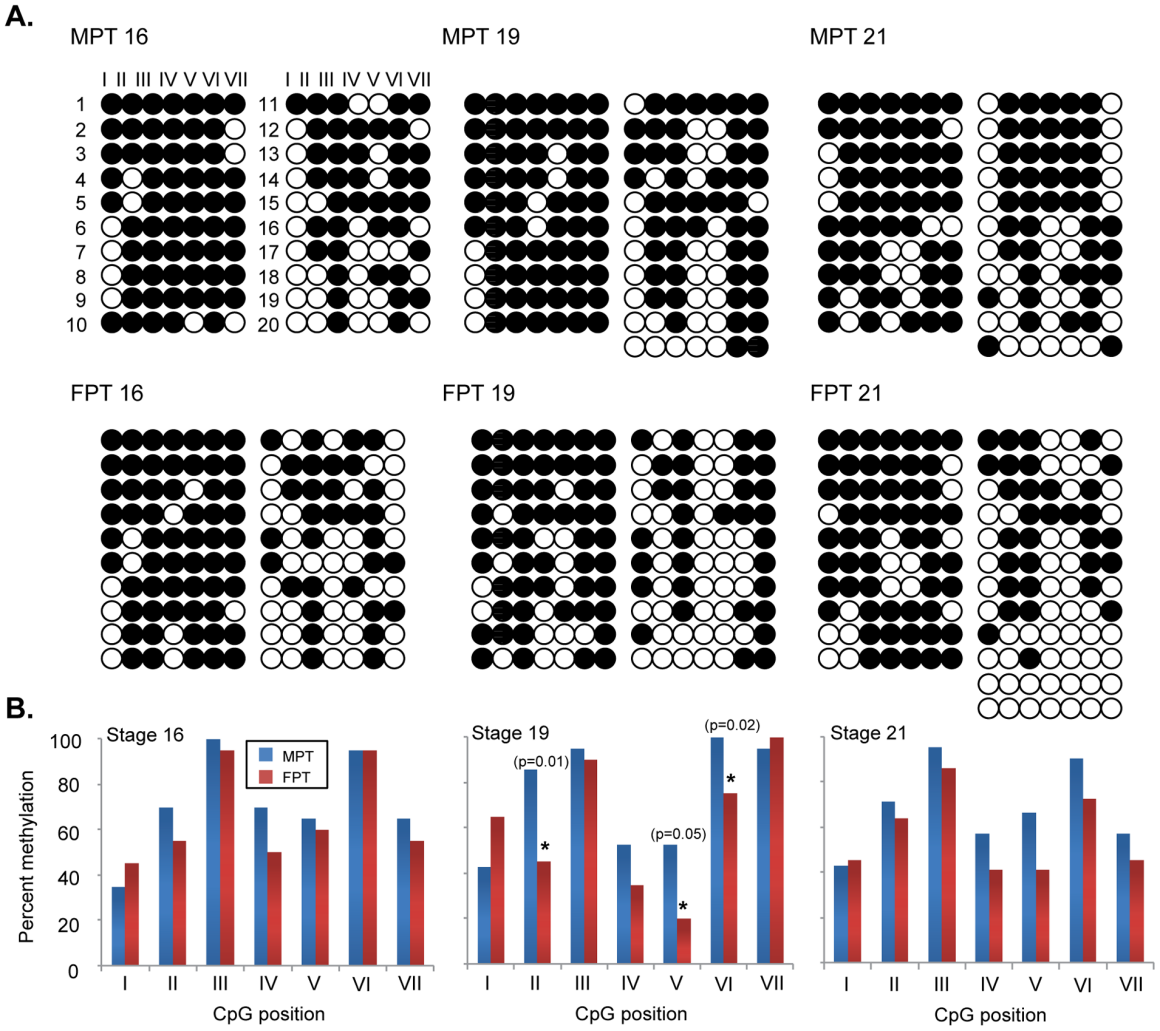
DNA methylation of the putative aromatase promoter at the individual CpG site in the gonads. (A) DNA methylation status in clones from the gonads at embryonic stage 16, 19, and 21 at MPT and FPT. Each row represents a single clone (1–20∼22 clones/stage/temperature) and a column represents the position of CpG (I – VII). Open circles: unmethylated CpG sites. Closed circles: methylated CpG sites. (B) Percent DNA methylation at the individual CpG site (I – VII) of the putative aromatase promoter at stage 16, 19, and 21 in the slider gonad. Asterisk (*) is statistically significant (p<0.05) according to the Fisher's exact test. MPT: male-producing temperature. FPT: female-producing temperature.

**Figure 6:**
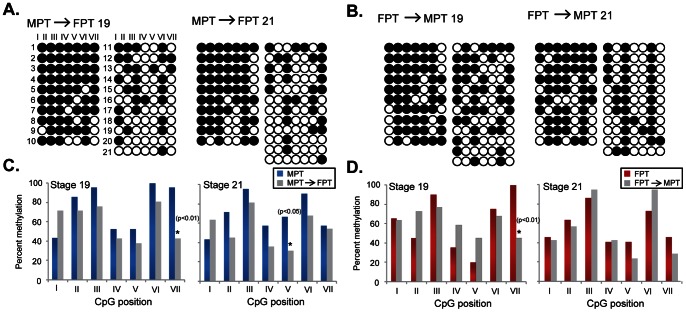
DNA methylation of the putative aromatase promoter at the individual CpG site in temperature-shifted gonads. At embryonic stage 16, eggs are shifted to the opposite temperature, MPT to FPT (A, C) and FPT to MPT (B, D). (A, B) DNA methylation status in clones from the gonads at embryonic stage 19 and 21 after temperature-shift. Each row represents a single clone (1–21∼22 clones/stage/temperature) and a column represents the position of CpG (I – VII). Open circles: unmethylated CpG sites. Closed circles: methylated CpG sites. (C, D) Percent DNA methylation at the individual CpG site (I – VII) of the putative aromatase promoter at stage 19 and 21 in the slider gonad. Asterisk (*) is statistically significant (p<0.05) according to the Fisher's exact test. MPT: male-producing temperature. FPT: female-producing temperature.

Since DNA methylation patterns are often correlated to the particular CpG sites within or near TFBSs that, in return, affect the binding of transcription factors [35], we next examined the methylation levels at the individual CpG site. At stage 19, three CpG positions at II, V, and VI exhibited different methylation patterns at the two incubation temperatures (Figure 5A, B). The CpG position II was located between FOX and SF1/ERE binding sites and the CpG position V and VI were just before and after the TATA box respectively (Figure 3). Regardless of the overall lower level of global methylation at FPT compared to MPT at stage 21, no individual CpG site significantly differed in methylation level by two incubation temperatures (Figure 5A, B). In temperature-shifted gonads, CpG position VII at stage 19 and position V at stage 21 were significantly lower at MPT→FPT than at MPT (Figure 6A, B). In the FPT→MPT treatment, the DNA methylation level at CpG position VII at stage 19 was significantly lower compared to the control FPT; however, none of other individual sites were differently methylated by incubation temperature (Figure 6A, B).

## Discussion

Aromatase is an essential factor for gonad determination and differentiation in non-mammalian vertebrates. Most studies to date have been limited to the gene expression profiles in differentiating gonads. In this study, we identified for the first time in TSD species the temperature-dependent epigenetic signatures within the gonad-specific promoter of the aromatase gene. In most mammals, birds, and amphibians, the aromatase gene has multiple TSS driven by tissue-specific promoters producing unique 5′-untranslated regions that are alternatively spliced along with a common coding region [36]–[39]. In fish, the tissue-specific aromatase transcripts, mostly studied in ovary and brain, originate from two or more different homologous genes [40]–[42]. Whether the aromatase gene of the red-eared slider turtle has multiple tissue-specific TSS or homologous genes is not known. However, the TSS in developing ovaries identified in the current study is likely gonad-specific according to previous observations in different species. This study also led to the discovery of the 1754 bp length of intron at +265 bp relative to the TSS. The sequence has been submitted to GenBank along with the 5′-flanking region (GenBank accession no. KC554066).

Our prediction for TFBSs within the 5′-flanking region of the aromatase revealed potential *cis*-regulatory mechanisms of aromatase gene expression. We found several SF1 sites located within the 500 bp upstream region of the translation start codon (Figure 2). SF1 is an orphan nuclear receptor essential for the regulation of reproduction-related genes and exhibits a coordinated gene expression pattern with aromatase in developing ovaries in several species [13], [43]–[45]. Others have shown that SF1 stimulates aromatase promoter activity both *in vivo* and *in vitro* [46]–[48]. We found one SF1 site located within 150 bp upstream of the gonad-specific TSS in slider aromatase; this same site is also observed in other species and thus is highly conserved (Figure S2). The slider aromatase also had another SF1 site, specific only to this species, just after the TSS (Figure S2). EREs, potential binding sites for ER, were also found in the 5′-flanking region of the slider aromatase (Figure 2). Accordingly, ERE is commonly observed within the ovarian-specific aromatase promoter in fish and amphibians [49]–[51]. We previously noted that administration of estrogen to eggs incubated at MPT increased gonadal aromatase expression, whereas blocking endogenous estrogen at FPT suppressed the aromatase expression in the red-eared slider (unpublished data). This finding suggests that estrogen has a positive feedback to aromatase expression at the transcriptional level during gonad development. The binding sites for FOX and DM exhibited distinct patterns that were clustering closely (i.e., a multiple binding sites within 100 bp) on the 5′-flanking region (Figure 2). Both transcription factors FoxL2 and Dmrt1 can directly regulate the expression of the aromatase gene in a positive and negative manner, respectively, as shown in both *in vivo* and *in vitro* studies [52]–[54]. Furthermore, the expression of FoxL2 and Dmrt1 genes are mutually exclusive in bipotential gonads, suggesting another level of complementary of gene regulation in the determination of ovary vs. testes [27], [55], [56].

We have shown previously that the expression of aromatase in gonads was suppressed at both incubation temperatures at stage 16 but at FPT rapidly increased thereafter [13]. Similarly, we observed no temperature difference in methylation level at stage 16, but significantly lower levels of methylation at FPT at stages 19 and 21 (Figure 4A). This suggests that low level of DNA methylation at the promoter region is responsible for FPT-specific increase in aromatase expression. Further, this temperature-specific pattern of methylation seems to be established between stage 16 and 19 (Figure 4A). Our temperature-shift treatment MPT→FPT indicated that FPT signal after stage 16 was sufficient to allow demethylation at the aromatase promoter (Figure 4C). Interestingly, the temperature-shift FPT→MPT did not result in an increase of methylation level (Figure 4D). This indicates that the demethylation is a temperature sensitive process, however, the initiation of methylation is independent from temperature signal after stage 16.

We further investigated if any individual CpG site is specifically methylated to predict the potential interaction of transcription factors to the promoter region. Our study showed that a CpG site located between FOX and SF1sites was less methylated at FPT than MPT at stage 19 (Figure 5). FoxL2 is one of the earliest ovarian markers that is expressed in differentiating gonads across species [57]–[59]. *In vitro* studies show that FoxL2 along with SF1 can enhance the expression of aromatase by directly interacting with the forkhead binding site of the promoter [52], [53]. Our finding suggests that a low methylation level may allow FoxL2 to bind to this site at FPT, which results in transcriptional activation of the aromatase. We also found that two CpG sites located just before and after the TATA box had a significantly low level of methylation at FPT (Figure 4). The TATA box is a target sequence for the TATA box-binding protein and therefore, is a site for RNA polymerase II recruitment [60], [61]. Our data indicates that the differential methylation signatures by incubation temperature nearby the TATA box may be responsible for the assembly of the transcription initiation complex. Navarro-Martin and others observed that an exposure of sea bass embryos to high temperature, which correlated to a male-biased sex ratio, leads to an increase in DNA methylation at the aromatase promoter region [22]. Furthermore, they report differentially methylated CpG sites dictated by incubation temperature near the Fox binding site and TATA box within the promoter. Despite little sequence similarities between slider and sea bass aromatase promoters, our results are consistent with their observations, which further confirms that temperature alters the pattern of DNA methylation at the specific sites of CpG within aromatase promoter in the gonads. It is interesting that differential DNA methylation patterns at the individual CpG sites disappeared at stage 21 (Figure 5), regardless of the continuous increase in gene expression at FPT [13]. In mammals, the developmental gene expression is, at least partially, regulated by a repeat of *de novo* methylation and demethylation of the regulatory regions [62]–[64]. It is possible that the DNA methylation pattern observed at the individual CpG site is a transitory mark for the initiation of aromatase transcription whereas the maintenance of transcription at following stages may be mediated via different mechanisms.

Although the overall DNA methylation level was decreased in MPT→FPT gonads, we did not find a consistent pattern of DNA methylation at the CpG position II, V, and VI in our temperature-shift experiments (compare Figure 5 to Figure 6A). These observations indicate that temperature-shift at stage 16 is not sufficient to establish the DNA methylation pattern specific to the CpG sites. It is possible that epigenetic marks, not limited to the DNA methylation or on aromatase gene, may already be established in gonads before stage 16 but after stage 16 only the level of methylation can be affected. We also cannot rule out the possibility that the methylation percentage is limited by the number of colonies examined in the sub-cloning technique. It is worth noting that the CpG position VII, located after the gonad-specific TSS, had significantly lower methylation in the both temperature-shifts (Figure 6A, B). We do not currently have an explanation for this observation, although methylation at this CpG position seems to be specific to the temperature-shift treatment as it was not observed in gonads at constant temperature. Although the temperature effect on the level of DNA methylation has been studied mostly in plants [65], [66], it is still unclear how temperature affects the DNA methylation in non-mammalian vertebrates. It is most likely that dynamic epigenetic changes in a particular set of genes occur during gonad determination. The current study of epigenetic profiles of the aromatase promoter marked by temperature will provide further insight in the process of TSD system and ultimately help to understand the mechanisms underlying developmental plasticity.

## Materials and Methods

### Tissue collection

Freshly laid red-eared slider turtle eggs were obtained from Clark Turtle Farm (Hammond, LA) and maintained in accordance with humane animal practices under IACUC protocol. The protocol was approved by IACUC (protocol # AUP-2011-00149) at the University of Texas at Austin. Eggs were stored at room temperature for 10 days to assess viability of the embryos and randomly assigned to incubators at either MPT (26°C) or FPT (31°C) with moistened vermiculite. Incubator temperatures were monitored daily with thermometers and HOBO data loggers (Onset Computer Corp., Bourne, MA). Eggs were incubated until Greenbaum's embryonic stage 16 [67], at which point embryonic gonads became responsive to ambient temperature, i.e. the beginning of the TSP. To determine the gonad-specific TSS, pooled gonads (n = 6 gonads/stage) from stage 16, 19, and 21 at FPT, corresponding to the beginning, middle, and end of TSP, respectively, were placed in 800 µl Trizol (Life Technologies, Grand island, NY), vortexed, and stored at −80°C until total RNA extraction. TSS was not examined at MPT because aromatase transcripts are typically undetectable at this temperature [13].

For the methylation assay, gonads (n = 10 gonads/temperature) at each temperature (MPT or FPT) were pooled at stage 16 as the base line control and snap-frozen in liquid nitrogen (LN_2_) for subsequent assays. Ten eggs were continuously kept at MPT or FPT while ten other eggs were shifted to the opposite temperature regime at stage 16. At stage 19 and 21, gonads (n = 10) from each treatment group were pooled, snap-frozen in LN_2_, and stored at −80°C until bisulfite treatment. These groups were MPT, FPT, MPT shifted to FPT (MPT→FPT), or FPT shifted to MPT (FPT→MPT). For the cloning of the 5′-flanking region of aromatase, tissues including the adrenal-kidney complex, liver, or head were separately collected from FPT hatchling, snap-frozen in LN_2_, and stored at −80°C for genomic DNA (gDNA) extraction.

### Cloning and sequence analysis of the 5′-flanking region

The 5′-flanking region upstream of the ATG start codon of the aromatase gene was cloned using the Genome Walker Universal Kit (Clontech, Mountain view, CA) according to the manufacturer's protocol. Briefly, frozen turtle tissues at hatching were grinded in LN_2_ using a mortar and pestle and digested in lysis buffer (10 mM Tris HCl pH 8.0, 0.1 M EDTA pH 8.0, 0.5% SDS) containing 10 µg of RNase A at 37°C for 1 hr. Proteinase K (50 µg) was added to the lysate for an overnight incubation at 55°C. gDNA was extracted by the conventional phenol-chloroform method and precipitated by ethanol (EtOH). The purity of the gDNA was confirmed by the presence of smear on a 0.6% agarose gel after a digestion with restriction enzyme DraI. gDNA extracted from the head had the most distinct smear after DraI digestion, therefore was utilized for subsequent assays.

To construct libraries, the gDNA was digested with restriction enzymes, DraI, EcoRV, PvuII, or StuI to produce four libraries. As a positive control, human gDNA supplied with the kit was digested with Pvu II. Digested gDNA was purified per the phenol-chloroform method and ligated with the Genome Walker^TM^ Adaptor. Two reverse primers, the aromatase gene specific primer (Arom GSP) 1 and Arom GSP2, were designed based on the known slider aromatase sequence of the first exon (GenBank accession no. AF178949). Primer sequences and locations are indicated in Table 1 and Figure S3. All primers in this study were purchased from Integrated DNA technologies (IDT, Coralville, IA). Each library was amplified with Arom GSP1 and Adaptor specific primer (ASP) 1 provided with the kit using the Advantage 2 Polymerase Mix (Clontech) under the following PCR conditions: 7 cycles of 25 sec at 94°C and 3 min at 72°C followed by 32 cycles of 25 sec at 94°C, 3 min at 67°C, and 7 min at 67°C. The PCR products were then diluted 1∶50 with deionized H_2_O to perform the second round of nested PCR using Arom GSP2 and ASP2 primers under the following PCR conditions: 5 cycles of 25 sec at 94°C and 3 min at 72°C, 20 cycles of 25 sec at 94°C, 3 min at 67°C, and 7 min at 67°C. The second PCR products were visualized on a 1.5% agarose gel, extracted using a gel extraction kit (Qiagen, Germantown, MD), and cloned in a pGEM-T vector (Promega, Madison, WI) for sequencing. Each library produced different sizes of PCR products (data not shown) that were subsequently aligned for the identification of the novel 5′-flanking region sequence of aromatase. Additional Arom GPS reverse primers (sequences not shown) were designed based on this 5′-flanking region to repeat the PCR and sub-cloning processes on the existing gDNA libraries until approximately 4 kb of the newly identified 5′-flanking region was identified and sequenced. A whole 5′-flanking region was generated by PCR and eight clones were aligned for the best sequence prediction. The sequenced data was submitted to GenBank (accession no. KC554066). The TFBSs for the vertebrate steroidogenic factor 1 (SF1), estrogen response elements (ERE), fork head domain factors (FOX), DM domain-containing transcription factor (DM), sox/SRY-sex/testis determining and related HMG box factors (SOX), and heat shock factor (HEAT) on the 5′-flanking region of aromatase were detected with MatInspector (Genomatix, Munich, Germany). The CpG islands within the 5′-flanking region were examined using CpG Plot (European Bioinformatics Institute, Cambridge, UK).

**Table 1:** Primer sequences to identify the 5′-flanking region of the aromatase gene and gonad-specific transcription start sites in the red-eared slider turtle.

Primer name	Direction	Sequence (5′–3′)	Length (bp)
Arom GSP 1	Reverse	CAC GCT GGT GAT ATT GGA ATG CAT CAG	27
Arom GSP 2	Reverse	CAC CTT TAA TCG GTA CCT TGC AAC CCC	27
Arom GSP 3	Reverse	ATA GTT GCA GGC ATT TCC CAT TCC CAT C	28
Arom GSP 4	Reverse	ATG CAT GCC AAT GCA CTG TAA CCC	24
Arom GSP 5	Reverse	TCC TCC AAT CTG TCC AGA TGG TCT	24

Arom GSP  =  Aromatase gene specific primer.

### Sequence homology analysis

Approximately 4 kb of the newly identified 5′-flanking region of the slider aromatase sequence was compared with the 5′ upstream region of known aromatase sequence in other species (GenBank accession no.) including the *alligator* (AY029233), *chicken* (D50335), *quail* (D50336), and *zebra finch* (AH008871) using PIPMaker [24]. Because the availability of 5′ flanking region of the alligator aromatase was limited in GenBank database, additional 4 kb upstream region was extracted from the recently assembled genome [68]. TFBSs of the promoter regions among these species were analyzed with MatInspector and aligned according to the reported gonad- or brain-specific TSS of the aromatase gene [32], [33].

### Identification of gonad-specific transcription start site

To identify gonad-specific TSS in the 5′-flanking region of aromatase, we conducted a modified RNA ligase-mediated rapid amplification of cDNA ends (RLM-5′RACE) described by Bohm et al. [69]. RLM-5′RACE is able to accurately determine the TSS by tracing the 5′cap of the mRNAs whereas a conventional 5′ RACE does not trace the 5′cap, therefore cannot distinguish degraded mRNA messages from complete messages (compare A to B in Figure S1). Briefly, total RNA was extracted from pooled gonads at embryonic stage 16, 19, and 21 at FPT (n = 6/stage) with Trizol according to the manufacturer's protocol. Five microgram of total RNA was treated with DNA-Free Turbo DNase I (Life technologies). DNase I and all subsequent enzymatic reactions were followed by phenol:chloroform:isoamyl alcohol (50∶49∶1; Sigma-Aldrich, S. Louis, MO) extraction to inactivate the enzymes, and the RNA was EtOH precipitated in a presence of 10 unit of RNase inhibitor, RNaseOUT (Life technologies). Purified total RNA was dephosphorylated with 10 units of Calf Intestinal Alkaline phosphatase (CIP; NEB, Ipswich, MA) at 42°C for 2 hrs. After the purification, the 5′cap was removed with 0.5 units of Tobacco Acid Pyrophosphatase (TAP; Epicentre, Madison, WI) at 37°C for 1 hr. As a negative control, CIP-treated RNA from each stage was incubated in the absence of TAP. For the preparation of the double-stranded RNA oligo adaptor, DipromF forward primer containing T7 and T3 forward sequences (5′-TTA ATA CGA CTC ACT ATA GGG CCC AGA ATT AAC CCT CAC TAA AGG GA) and the reverse compliment sequence of DiPromF, RCDPromR reverse primer were designed. One microgram of each primer were incubated together with 10X T4 DNA ligase buffer (NEB) at 95°C for 5 min and placed at 65°C heat block which was then left at room temperature to naturally cool to the ambient temperature (approximately 26°C) for the slow annealing process.

Double-stranded DNA oligo was *in vitro*-transcribed to RNA oligo using mMESSAGE mMACHINE T7 kit (Life technologies) according to the manufacturer's protocol. The final concentration of RNA oligo after T7 transcription was approximately 50 µg. The 10 µM of the RNA oligo adaptor was ligated with decapped RNA using 5 units of T4 RNA ligase (NEB) at 37°C for 1.5 hrs. Ligated products were reverse transcribed with 200 units of SuperScript III reverse transcriptase (Life technologies) using 2 pmol of Arom GSP5 reverse primer (Table 1, Figure S3). The cDNA was amplified with DiPromF forward primer along with Arom GSP 4 reverse primer (Table 1, Figure S3) using Taq polymerase (Life technologies) under the following PCR conditions: 3 min at 94°C, 35 cycles of 45 sec at 94°C, 30 sec at 58°C, and 2 min at 72°C, and an additional extension for 10 min at 72°C. The second round of nested PCR was conducted with a 1∶50 dilution of the PCR product using the DiPromF primer and the Arom GSP3 primer (Table 1, Figure S3). The PCR products were visualized and extracted from a 1.5% agarose gel, sub-cloned into a pGEM-T vector, and the first nucleotide at the 5′end in each clone was confirmed by sequencing. None of the negative controls, not treated with TAP, produced a distinct band on the 1.5% agarose gel (data not shown). Approximately 22–28 clones from each stage were analyzed to determine gonad-specific-TSS in sequence analysis.

### DNA methylation analysis

DNA methylation status in the region of gonad-specific TSS (between −571 and +23 relative to the position of TSS counted as +1) of the slider aromatase gene was examined using EZ DNA Methylation-Direct kit (Zymo research, Irvine, CA). Ten pooled gonads taken from embryos at stage 16, 19, and 21 were incubated at MPT, FPT, MPT→FPT, and FPT→MPT. They were treated with Proteinase K and bisulfite and purified according to the manufacture's protocol. Purified gDNA were amplified using Advantage 2 Polymerase Mix with Arom Set5F primer, 5′- GGY GTT AGG TTT GAT GAA TTT ATG GTT TGG-3′ and Arom Set5R primer, 5′- ACA CCT TTA ATC RAT ACC TTA CAA CCC -3′ under the following PCR conditions: 1 min at 95°C, 35 cycles of 30 sec at 95°C, 1 min at 56°C, 1 min at 68°C, and 10 min at 72°C for 10 min. PCR products were gel purified and sub-cloned as described above. The average rate of bisulfite conversion was >99%, which was calculated by the number of converted cytosine that were adherent to non-guanines. The clones with less than 97% conversion rate were excluded from the analysis to avoid miscalling an incomplete bisulfite conversion as a legitimate DNA methylation. A total of 20–22 clones/stage/group were subjected to the sequence analysis.

### Statistical analysis

The ratio of methylated to unmethylated CpG at each position were compared between the treatment groups (MPT vs FPT, MPT vs MPT→FPT, or FPT vs FPT→MPT) by Fisher's exact test using R (The R project for Statistical Computing, Vienna, Austria). A p-value of less than 0.05 was considered to be statistically significant.

## Supporting Information

Figure S1 **Nucleotide position of gonad-specific transcription start sites (TSSs) of aromatase in the red-eared slider turtle.** TSSs were examined in total RNA from pooled gonads at embryonic stage 16, 19, and 21 at FPT (n = 6/stage) using (A) RNA ligase-mediated rapid amplification of cDNA ends (RLM-5′RACE) and (B) A conventional 5′RACE using SMART^TM^ RACE cDNA Amplification kit (Clontech). X-axis represents the distance in base-pairs from the translation start codon (ATG), and the y-axis represents the number of clones examined.
(TIF)

Figure S2 **Comparative analysis of the transcription factor binding sites (TFBSs) of the aromatase gene among species.** TFBSs at approximately 700 bp of the 5′-flanking regions including the translation initiation codon (ATG) and transcription start site (TSS) of the red-eared slider turtle and four other species were predicted using the MatInspector (Genomatix) and aligned relative to the gonad- or brain-specific TSSs. GenBank accession numbers of these species are as follows: *alligator* (AY029233), *chicken* (D50335), *quail* (D50336), and *zebra finch* (AH008871). Dashed lines indicate missing sequence information of the alligator aromatase in the GenBank. Colored marks represent TFBSs with a 5′ to 3′ (above the line) or a 3′ to 5′ (below the line) orientation relative to the TSS. Color codes for the transcription factors are as follows: pink: SF1 (vertebrate steroidogenic factor 1), light green: ERE (estrogen response elements), ocher: FOX (fork head domain factors), dark green: DM (DM domain-containing transcription factor), purple: SOX (Sox/SRY-sex/testis determining and related HMG box factors), and dark blue: HEAT (heat shock factors). TATA: TATA box. Horizontal black arrow: TSS. Reverse triangle: ATG. Solid vertical arrows: conserved SF1/ERE and Sox binding sites. Dashed vertical arrows: conserved SOX binding sites.
(TIF)

Figure S3 **Relative positions of the designed primers in** **Table 1** **.** Black line: known aromatase cDNA sequence of the red-eared slider turtle (GenBank accession no. AF178949). Orange line: unknown 5′-flanking region. Black arrows: aromatase gene-specific reverse primer (GSP) positions. A translation start codon, ATG was previously identified in the red-eared slider turtle [23].
(TIF)
