# Supplementary figures and images for: Epigenetic Control of Gonadal Aromatase (cyp19a1) in Temperature-Dependent Sex Determination of Red-Eared Slider Turtles
Source: PLoS One. 2013 Jun 7;8(6):e63599. doi: 10.1371/journal.pone.0063599 (PMC3676416; doi:10.1371/journal.pone.0063599)

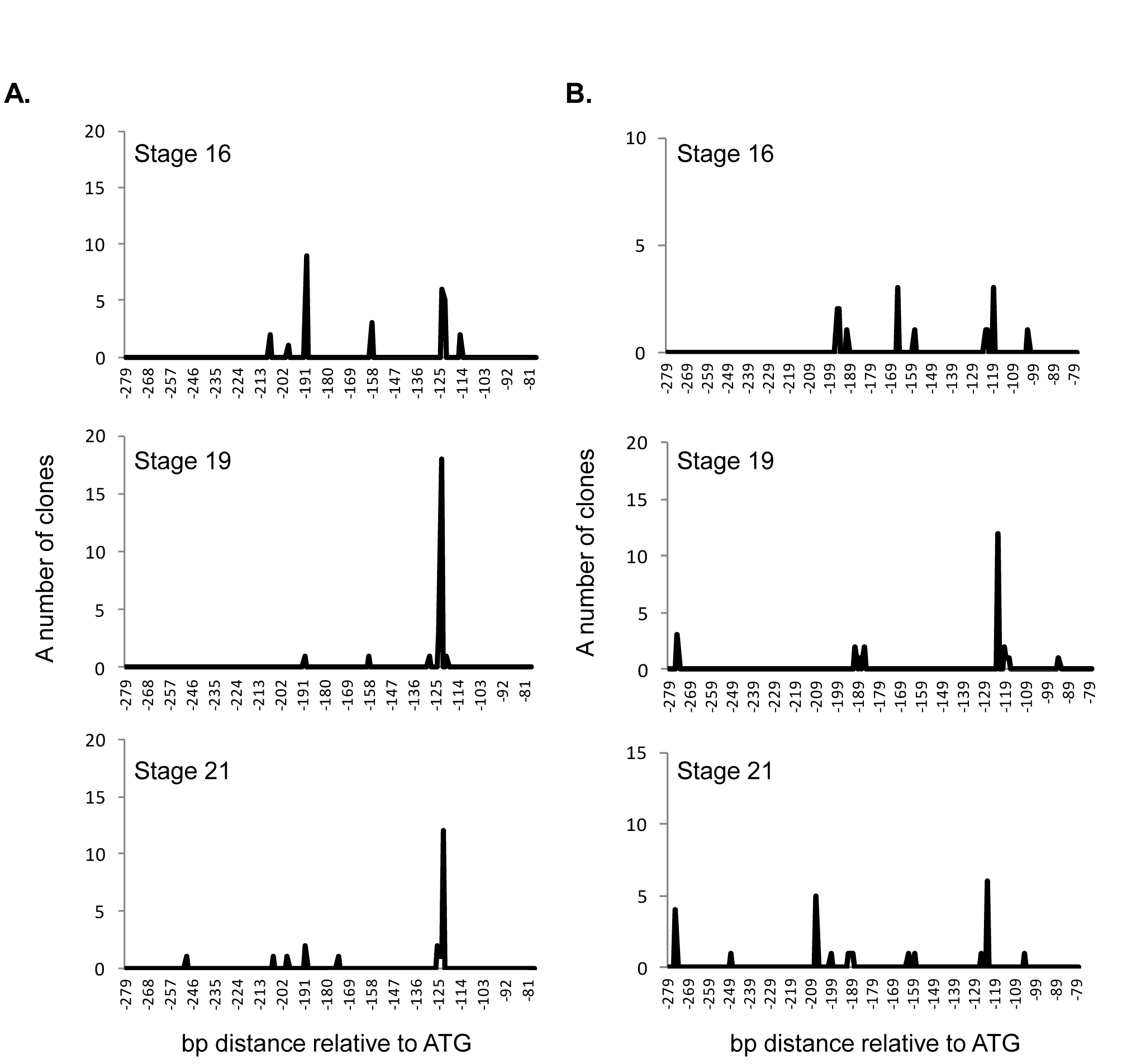

Supplement: Figure S1 — Nucleotide position of gonad-specific transcription start sites (TSSs) of aromatase in the red-eared slider turtle. TSSs were examined in total RNA from pooled gonads at embryonic stage 16, 19, and 21 at FPT (n = 6/stage) using (A) RNA ligase-mediated rapid amplification of cDNA ends (RLM-5′RACE) and (B) A conventional 5′RACE using SMARTTM RACE cDNA Amplification kit (Clontech). X-axis represents the distance in base-pairs from the translation start codon (ATG), and the y-axis represents the number of clones examined. (TIF) [file pone.0063599.s001.tif]

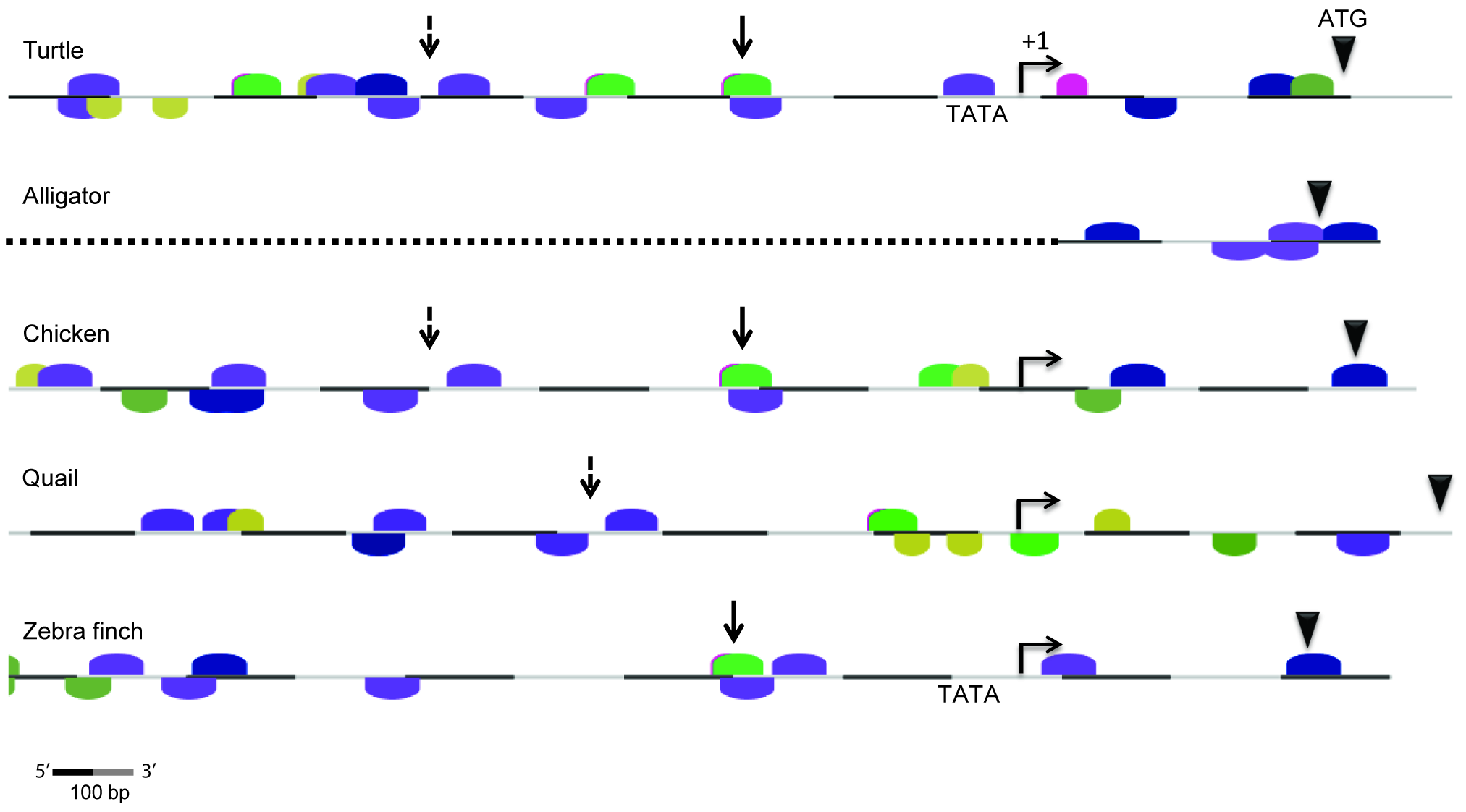

Supplement: Figure S2 — Comparative analysis of the transcription factor binding sites (TFBSs) of the aromatase gene among species. TFBSs at approximately 700 bp of the 5′-flanking regions including the translation initiation codon (ATG) and transcription start site (TSS) of the red-eared slider turtle and four other species were predicted using the MatInspector (Genomatix) and aligned relative to the gonad- or brain-specific TSSs. GenBank accession numbers of these species are as follows: alligator (AY029233), chicken (D50335), quail (D50336), and zebra finch (AH008871). Dashed lines indicate missing sequence information of the alligator aromatase in the GenBank. Colored marks represent TFBSs with a 5′ to 3′ (above the line) or a 3′ to 5′ (below the line) orientation relative to the TSS. Color codes for the transcription factors are as follows: pink: SF1 (vertebrate steroidogenic factor 1), light green: ERE (estrogen response elements), ocher: FOX (fork head domain factors), dark green: DM (DM domain-containing transcription factor), purple: SOX (Sox/SRY-sex/testis determining and related HMG box factors), and dark blue: HEAT (heat shock factors). TATA: TATA box. Horizontal black arrow: TSS. Reverse triangle: ATG. Solid vertical arrows: conserved SF1/ERE and Sox binding sites. Dashed vertical arrows: conserved SOX binding sites. (TIF) [file pone.0063599.s002.tif]

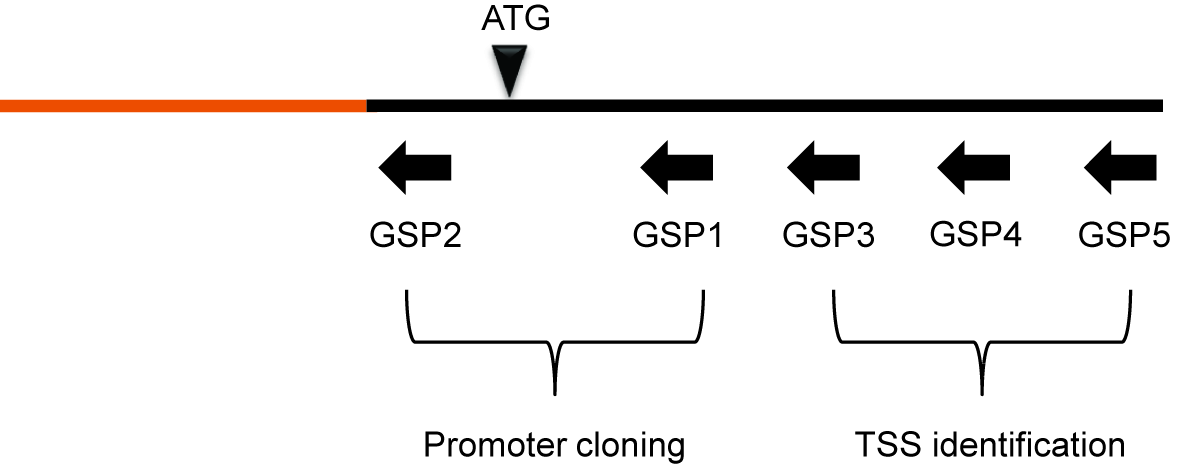

Supplement: Figure S3 — Relative positions of the designed primers in Table 1 . Black line: known aromatase cDNA sequence of the red-eared slider turtle (GenBank accession no. AF178949). Orange line: unknown 5′-flanking region. Black arrows: aromatase gene-specific reverse primer (GSP) positions. A translation start codon, ATG was previously identified in the red-eared slider turtle [23]. (TIF) [file pone.0063599.s003.tif]
